# Restriction Factors: From Intrinsic Viral Restriction to Shaping Cellular Immunity Against HIV-1

**DOI:** 10.3389/fimmu.2018.02876

**Published:** 2018-12-06

**Authors:** Marta Colomer-Lluch, Alba Ruiz, Arnaud Moris, Julia G. Prado

**Affiliations:** ^1^IrsiCaixa AIDS Research Institute, Germans Trias i Pujol Research Institute, Universitat Autonoma de Barcelona, Badalona, Spain; ^2^Sorbonne Université, INSERM U1135, CNRS ERL 8255, Centre d'Immunologie et des Maladies Infectieuses (CIMI-Paris), Paris, France

**Keywords:** restriction factors, HIV-1, innate immunity, adaptive immunity, virus, degradation pathways, immunotherapies

## Abstract

Antiviral restriction factors are host cellular proteins that constitute a first line of defense blocking viral replication and propagation. In addition to interfering at critical steps of the viral replication cycle, some restriction factors also act as innate sensors triggering innate responses against infections. Accumulating evidence suggests an additional role for restriction factors in promoting antiviral cellular immunity to combat viruses. Here, we review the recent progress in our understanding on how restriction factors, particularly APOBEC3G, SAMHD1, Tetherin, and TRIM5α have the cell-autonomous potential to induce cellular resistance against HIV-1 while promoting antiviral innate and adaptive immune responses. Also, we provide an overview of how these restriction factors may connect with protein degradation pathways to modulate anti-HIV-1 cellular immune responses, and we summarize the potential of restriction factors-based therapeutics. This review brings a global perspective on the influence of restrictions factors in intrinsic, innate, and also adaptive antiviral immunity opening up novel research avenues for therapeutic strategies in the fields of drug discovery, gene therapy, and vaccines to control viral infections.

## Introduction

Restriction factors are host cellular proteins contributing to the frontline defense against viral infections. Restriction factors recognize and interfere with specific steps of the replication cycle of viruses, thereby blocking infection. They are generally interferon (IFN)-inducible and their inherent features, such as constitutive expression in different cell types, self-sufficient activity, and rapidity of action, confer a potent and early restriction of viruses (1). So far, more than nine groups of cellular restriction factors have been identified that inhibit Human Immunodeficiency Virus type 1 (HIV-1), and other primate lentiviruses, including the classical and well-documented APOBEC3G, SAMHD1, Tetherin/BST-2, and TRIM5α (2–10), and those of more recent characterization MX-2, SERINC3/5, IFITMs, Schlafen 11, and MARCH2/8 (11–16). The continuous adaptation of HIV-1 to the pressure exerted by the antiviral activities of restriction factors underscores the importance of restriction factors in controlling viral infections.

A tight regulation of innate and adaptive immune responses is required to counteract infections. During the acute phase of viral infections, pro-inflammatory cytokine storms contribute to controlling viral replication. These cytokine secretions are initiated in response to pattern recognition receptor (PRR) engagement by viral molecules (or pathogen-associated molecular patterns, PAMPs) and the recruitment at the site of entry of innate immune cells including natural killer (NK) cells, macrophages, dendritic cells (DCs), and other phagocytic cells. Innate immune cells, especially DCs, further orchestrate the priming of cells involved in adaptive immunity, meaning CD4+ T helper cells, CD8+ cytotoxic T lymphocytes (CTLs), and B cells (17). In particular, during HIV-1 infection, a robust CTL response has been linked to a reduction of HIV-1 viral loads and a delay in disease progression (18–26).

Beyond their intrinsic antiviral activity, recent evidence demonstrates that several restriction factors also participate in the modulation of HIV-1-specific cellular adaptive immunity, highlighting the multifaceted nature of these proteins. The connections of restriction factors with the cellular degradation machinery and its pathways suggest that common mechanisms might be shared by restriction factors to promote anti-HIV-1 cellular immunity. Here, we provide an overview of the cell-intrinsic HIV-1 restriction activity of APOBEC3G, SAMHD1, Tetherin, and TRIM5α proteins, focusing on their complex interplay with innate and adaptive immune responses in the context of HIV-1 infection. Besides, we briefly comment on the potential of a similar interplay in the case of more recently discovered anti-HIV-1 factors. In our view, a better understanding of the molecular interactions between restriction factors, viruses, and the protein degradation machinery might help in developing novel therapeutic strategies to enhance innate and adaptive immune responses against viral infections such as HIV-1.

## Restriction Factors: Intrinsic Antiviral Activity and Modulation of Innate and Adaptive Immunity

### APOBEC3G

APOBEC3G (A3G) proteins belong to the AID/APOBEC family (apolipoprotein B mRNA editing enzyme, catalytic polypeptide-like) of cytidine deaminase enzymes. AID/APOBEC family members restrict a broad range of viruses including hepatitis B virus (HBV) as well as endogenous and pathogenic retroviruses (27–30). Interestingly, it was the interaction of the HIV-1 accessory protein Vif with A3G that led to the identification of A3G as a restriction factor (31). Upon HIV-1 infection, A3G and other APOBEC family members, such as APOBEC3F (A3F), are encapsidated into budding virions. In newly infected cells, during the reverse transcription of the viral RNA, A3G and A3F catalyze cytosine-to-uracil deamination in the nascent viral DNA. As a consequence, the proviral DNA harbors a high frequency of G-to-A hypermutations leading to the introduction of amino acid substitutions and premature STOP codons. Transcriptional activation of A3G-edited provirus yields to the production of defective proteins and non-functional viral particles, resulting in a strong inhibition of HIV-1 replication (7, 8, 32). Although the capacity to inflict G-to-A mutations has been considered as the central mechanism of A3G- and A3F-mediated restriction, A3G and A3F also exert deaminase-independent viral restriction (33, 34).

The HIV-1 Vif protein has evolved to antagonize A3G antiviral activity as well as other APOBEC family members that restrict viral infection (34, 35). In brief, Vif binds to A3G promoting the recruitment of the ElonginB/C-Cullin-5 E3 ubiquitin ligase complex leading to A3G poly-ubiquitination and proteasomal degradation, which results in a lower rate of A3G incorporation within the newly produced virions (36–38).

A3G is the most well-defined anti-HIV-1 protein of the APOBEC3 (A3) group. In humans, the A3 group consists of seven enzymes largely distributed in different cells and tissues, which contribute to DNA/RNA metabolism and cellular maintenance through their DNA/RNA deamination activities (32, 39, 40). Specifically, A3G and A3F are highly abundant in multiple cell types (41), but their expression in immune cells, predominantly in activated T cells, monocytes, macrophages, and mature DCs, strongly suggests that A3G and A3F exert diverse functions in immunity (42). On the one hand, various inflammation mediators like IFN-α increase A3G expression in monocytes, macrophages, and plasmacytoid dendritic cells (pDCs), while IFN-γ and IFN-β enhance A3G expression in macrophages (41, 43, 44), indicating that A3G and A3F are encoded by IFN-stimulated genes and suggesting that these enzymes play a central role in innate antiviral immunity. In fact, the expression of A3G is induced by pathogen sensors such as Toll-like receptors (TLRs), as well as by cytokines such as IL-2, IL-7, IL-15, and IL-27 (45).

**Figure 1:**
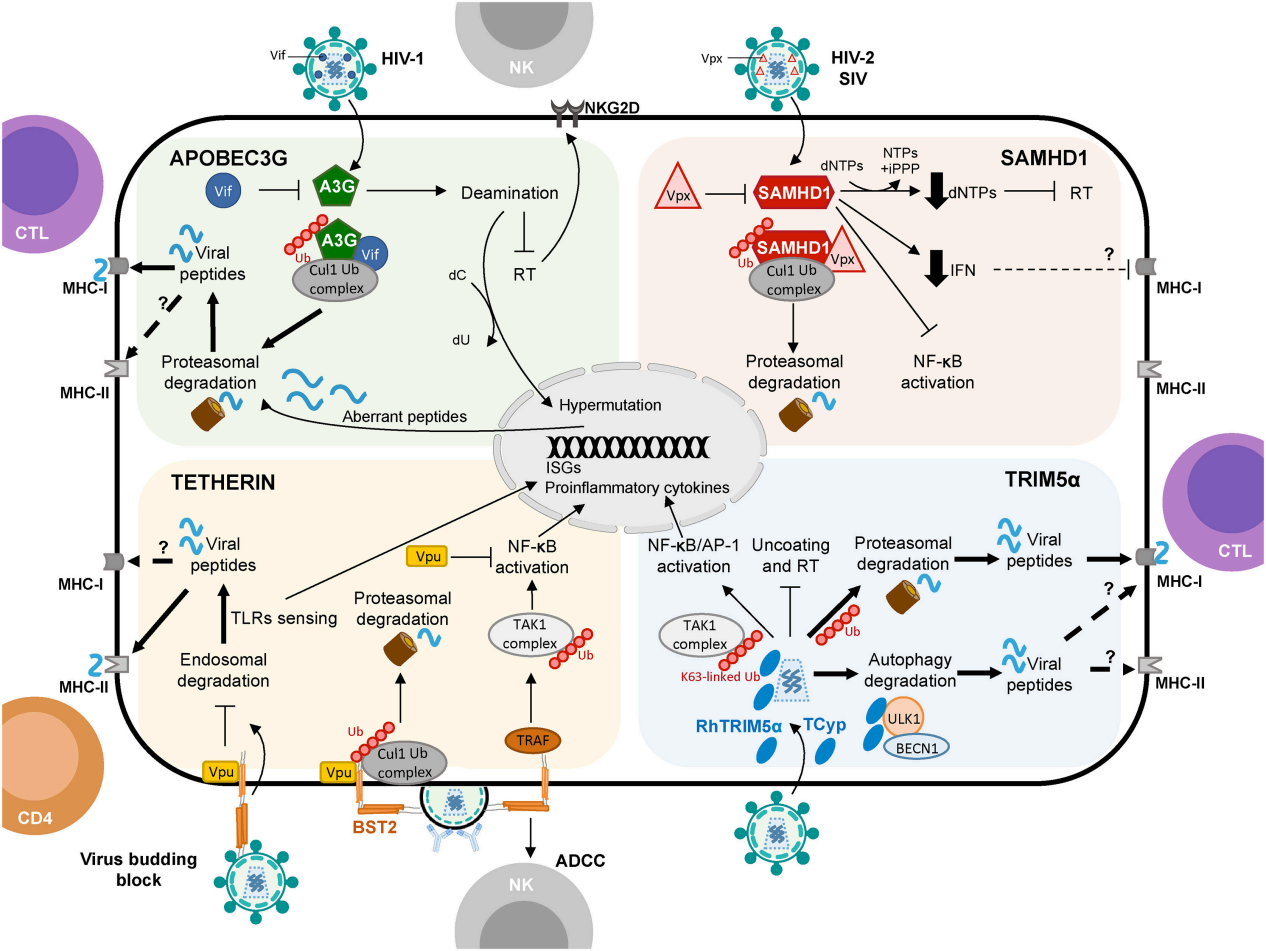
Schematic representation of APOBEC3G, SAMHD1, Tetherin, and TRIM5α proteins shaping cellular innate and adaptive immune responses against viruses with a focus on HIV-1 infection. Restriction factors link to protein degradation pathways and their connection to CD4+ T cell and CTL responses are depicted by thick arrows. Question marks (?) and broken arrows represent questions and unresolved pathways. Restriction factors respective counteracting proteins are also shown.

On the other hand, A3G itself also promotes innate and adaptive immunity. In particular, A3G-mediated cytidine deamination is sensed by the cellular DNA repair machinery driving the induction of stress responses and the activation of NK cells (46). Also, mouse APOBEC3 (mA3) was reported to act as a modulator of adaptive immunity eliciting both CTL responses and the generation of neutralizing antibodies (nAbs) against Friend retrovirus infection (47–49). However, how mA3 exacerbates CTL and nAbs production is not well-defined. In human cells, Casartelli et al. demonstrated that the editing activity of A3G favors the generation of HIV-1 antigenic peptides (epitopes) by infected cells leading to enhanced activation of HIV-1-specific CTL responses (50). In brief, in CD4+ T cells, the A3G-mediated HIV-1 restriction was associated with enhanced activation of HIV-1-specific CTL responses. The mechanism proved to be dependent on the A3G-mediated editing of the viral genome since A3G mutants with impaired editing activity failed to induce CTL activation. Furthermore, introducing a premature STOP codon in HIV-1 genome, mimicking A3G editing, led to higher CTL activation most likely due to the generation of additional viral epitopes processed by proteasomes and presented by MHC class-I (MHC-I) molecules (50, 51) (Figure 1). These results were further corroborated in the context of A3G (and A3F) expression in DCs and CTL activation, suggesting that in infected DCs, A3G- and A3F-mediated editing of viral genomes might enhance the capacity of DCs to prime antiviral CTL responses (52). Interestingly, although A3G editing activities can positively influence CTL activation, they can also negatively affect CTL responses by aiding to the emergence of CTL escape mutations (53, 54). The A3G-mediated effect on HIV-1 epitope presentation to CTLs appears to be beneficial or detrimental depending on the HLA allotype of the subject (55). Thus, a precise calibration between A3G-derived antigen generation and incorporation of epitope mutational changes is fundamental to contribute to the activation of specific CTL responses against HIV-1 infected cells via A3G. Remarkably, in patients under effective antiretroviral treatment, CTL recognition of epitopes derived from A3G-mediated mutated polypeptides and expressed by defective proviruses probably shapes the repertoire of latently infected cells (56).

Interestingly, studies of A3G in rhesus macaques revealed that upon immunization with SIV antigens and CCR5 peptides linked to the HSP70 protein there was a progressive increase in A3G expression in memory CD4+ T cells (57, 58), eliciting protection against the virus through induction of innate and adaptive immunity. Similarly, immunization of rhesus macaques with recombinant HLA constructs, linked with HIV-1 and SIV antigens and HSP70, resulted in upregulation of A3G in CD27+ memory B cells (59), which might be associated with a protective effect against infection. In humans, B cells express A3G that has been postulated to be transmitted via exosomes to targets of HIV-1 infection to exert its antiviral activity (31, 32, 60).

### SAMHD1

SAMHD1 (Sterile Alpha Motif and Histidine Aspartate domain-containing protein 1) is a deoxynucleotide triphosphohydrolase that impairs HIV-1 reverse transcription by reducing the pool of cellular dNTPs (5, 61, 62). Upon HIV-1 infection of CD4+ T cells, the HIV-1 capsid is delivered into the cytoplasm allowing reverse transcription of the viral RNA into DNA, a step that is strictly dependent on the availability of the dNTPs. SAMHD1 hydrolyzes all four dNTPs to deoxynucleosides and inorganic triphosphate thus controlling the pool of cytosolic dNTPs. As a consequence, in myeloid cells, SAMHD1 prevents proviral DNA formation and HIV-1 replication (4–6). In addition to its dNTPase activity, SAMHD1 also exhibits an RNase activity that might as well participate in SAMHD1-mediated viral restriction, for instance by targeting viral RNA for degradation before RT occurs (63, 64). However, to what extent this RNase activity contributes to HIV-1 restriction remains an open question (63, 65, 66).

HIV-2 and certain simian immunodeficiency virus strains (SIVsm/SIVmac) encode an accessory protein, Vpx, to overcome SAMHD1 restriction (67). Vpx interacts with the C-terminal domain of SAMHD1 recruiting the Cullin-4 E3 ubiquitin ligase complex, which destines SAMHD1 for poly-ubiquitination and proteasomal degradation thus relieving SAMHD1-mediated retroviral blockade (4, 6, 68). While Vpx from HIV-2 and most SIVs efficiently oppose SAMHD-1, HIV-1 and its simian ancestor infecting chimpanzees (SIVcpz) lack Vpx and are unable to counteract SAMHD1 and are vulnerable to its action (4).

Some myeloid cells—monocytes, macrophages, and DCs—and CD4+ T cells ubiquitously express SAMHD1 (4, 6, 69), which regulates their cell cycle by controlling the availability of the dNTP pools. Apart from being the substrates of SAMHD1, dNTPs, mainly driven by the levels of dGTP, globally regulate and equilibrate SAMHD1 structural states (61). However, the high expression levels of SAMHD1 both in cells refractory to HIV-1 such as monocytes and quiescent lymphocytes, or in cells permissive to HIV-1 such as macrophages and activated lymphocytes, argues for a posttranslational regulation of SAMHD1 functions (70, 71). Indeed, the Cyclin-dependent kinase 6 (CDK6) coupled with cyclin D3 was shown to control CDK2-dependent SAMHD1 phosphorylation in proliferating cells (72, 73). The phosphorylation of SAMHD1 alleviates its capacity to hydrolyze dNTPs controlling the availability of dNTPs for cellular DNA synthesis during the cell cycle and reverse transcription of the viral RNA (72).

Given its ability to limit the intracellular dNTP pool, SAMHD1 is tightly linked to the mechanisms of cell-cycle progression, as dNTPs availability is crucial for cycling cells. In addition, SAMHD1 may avoid the accumulation of nucleotides that otherwise would trigger innate immune sensing leading to undesired IFN-I secretion and chronic inflammation (66). In fact, deficiency in SAMHD1 leads to increased IFN production, upon viral DNA sensing by cGAS, and innate immune activation causing autoimmune disorders in patients (74). This is illustrated by mutations in SAMHD1 described as the cause of some cases of the Aicardi-Goutières syndrome, a rare autoimmune disease characterized by an IFN-stimulated gene expression signature that resembles a congenital viral infection and overlaps clinically and biochemically with the systemic lupus erythematosus (75). Recently, Chen and colleagues also suggested that SAMHD1 may act as a negative regulator of the cell-intrinsic antiviral responses (76). They described, *in vitro* and *in vivo*, how SAMHD1 down-regulates innate antiviral immune responses and inflammation by actively inhibiting NF-κB activation (by reducing the phosphorylation of IκBα) and IFN-I induction (by reducing IKKε-mediated IRF7 phosphorylation).

Owing to the inefficient capacity of HIV-1 to infect DCs, mainly due to the restriction exerted by SAMHD1, it has been proposed that HIV-1 might evade innate sensing (4, 77). In this context, it has been shown that lentiviral transduction of SAMHD1 in myeloid cells prevents the induction of IFN responses as well as antigen presentation (78). The blockage of HIV-1 by SAMHD1 might lower the capacity of DCs and macrophages to detect cytosolic viral DNA preventing the activation of the cGAS/STING pathway for IFN production and innate antiviral immunity. Hence, SAMHD1 negative regulation of the innate immune response would be used by HIV-1 as a mechanism to bypass host innate immunity (66). However, this function of SAMHD1 is rather indirect and numerous reports demonstrated that HIV-1-infected DCs exhibit an intermediate maturation phenotype, suggesting that HIV-1 cryptic replication in DCs is sensed and engages intrinsic innate immune responses (52, 77, 79).

Few studies have addressed the potential role of SAMHD1 in connecting innate and adaptive immune responses in HIV-1 infection. Ayinde et al. evaluated the contribution of SAMHD1 in HIV-1 antigen presentation via MHC-I molecules by monocyte-derived DCs (80). The authors demonstrated that SAMHD1 antiviral activity hampers HIV-1 replication while Vpx-mediated depletion of SAMHD1 augments the presentation of viral antigens by DCs, which leads to the activation of HIV-1-specific CTL responses and the killing of infected DCs (80) (Figure 1). Similarly, SAMHD1 retroviral restriction has been associated with defects in virus-specific CD8+ T cell responses in a lentiviral-infected mice model (78).

Several studies suggested that SAMHD1 may play a role in HIV-1 pathogenesis and disease progression, albeit conclusive evidence of a protective role are still lacking. While some authors described an association between the reduction of SAMHD1 activity in DCs with the capacity to naturally suppress viral replication in the absence of antiretroviral treatment (in so-called elite controllers) (81), others reported an up-regulation (82) or absence of differences in SAMHD1 expression levels in elite controllers as compared to control groups (83).

Nonetheless, it is interesting to note that SAMHD1 restriction activity might compete with the activation of innate and adaptive immune responses. This is in agreement with observations in autoimmune diseases and cancer where deregulation of dNTP levels and defects in nucleic acids metabolism impair cellular viability and trigger chronic stimulation of innate immune responses [reviewed in 66)].

### Tetherin/BST-2

Tetherin/BST-2 (Bone marrow stromal antigen 2) is an IFN-inducible transmembrane protein that potently anchors budding viral particles on the surface of infected cells, preventing the release of HIV-1 and other enveloped viruses (9, 10). This function is achieved by Tetherin's unique topology, with the presence of an N-terminal transmembrane domain and a C-terminal glycosyl-phosphatidylinositol group, which allows one end of the protein to be attached to the plasma membrane and the other to the viral envelope. The retained virions are then internalized and degraded via the endosomal/lysosomal pathway (84, 85).

Tetherin exerts antiviral activity against a broad spectrum of enveloped viruses. However, primate lentiviruses have evolved three different viral proteins to escape Tetherin restriction in a species-specific manner. The HIV-1 Vpu protein overcomes human Tetherin restriction by (i) promoting the poly-ubiquitination of its transmembrane domain inducing its proteasomal degradation, (ii) downregulating Tetherin's concentration on the cell surface, and (iii) sequestering Tetherin in endosomal compartments leading to its lysosomal degradation via a non-canonical autophagy dependent pathway (10, 86–92). In contrast, in HIV-2 the Env protein circumvents human Tetherin (93). In the case of most SIVs, the Nef protein targets a five amino acid sequence in the N-terminal cytoplasmic domain promoting simian Tetherin endocytosis and lysosomal degradation (94–96). Interestingly, human Tetherin lacks this five amino acid region conferring resistance to Nef antagonism.

Upon viral infection, most immune cells upregulate Tetherin expression, which has a short and a long isoform. The short isoform of Tetherin lacks 12 residues in its cytoplasmic tail and is resistant to Vpu-driven counteraction. Meanwhile, the longer version has been linked to the activation of the NF-κB pro-inflammatory signaling cascade (97, 98). In fact, the accumulation of tethered virions at the plasma membrane results in the activation of the NF-κB signaling pathway with the subsequent induction of pro-inflammatory responses (99–101), unveiling Tetherin's additional function as a viral sensor in innate immunity beyond its direct restriction activity. Note that Vpu abrogates Tetherin-mediated NF-kB activation (102). In addition, Tetherin seems to be involved as a negative regulator of innate immunity through an interaction with the immunoglobulin-like transcript 7 (ILT7) inhibitory receptor. In pDCs, the interaction of Tetherin with ILT7 impairs TLR signaling inhibiting IFN-I and proinflammatory cytokine production (103, 104).

Interestingly, Arias et al. elegantly demonstrated that Tetherin enhances the susceptibility of HIV-1 infected cells to antibody-dependent cellular cytotoxicity (ADCC) by introducing mutations in Vpu that prevent Tetherin antagonism. Consistently, RNA silencing of Tetherin expression decreased the susceptibility of HIV-1-infected cells to ADCC (105) (Figure 1). Remarkably, overexpression of BST-2 in response to IFN-α, but also to IFN-β and IL-27 that upregulate Env expression at the cell surface, sensitizes HIV-1-infected cells to elimination by ADCC (106). Thus, a Tetherin-mediated increased sensitivity of HIV-1-infected cells to ADCC may serve as a link between innate and adaptive immunity to augment the susceptibility of virus-infected cells to antibodies, with the potential to enhance other immune responses to control viral replication *in vivo*.

It has been proposed that Tetherin-mediated virus internalization might feed TLR containing compartments with viral PAMPs, thus enhancing TLR activation (107) but also providing antigens to compartments rich in MHC class-II (MHC-II) molecules leading to antigen presentation of viral peptides, thus affecting both innate and adaptive immune responses. In line with this hypothesis, Li et al. provided evidence that Tetherin promotes NK cell, and virus-specific CD4+ and CD8+ T cell responses in a mouse model of Friend retrovirus infection (108). Tetherin knockout mice showed weaker antiviral responses compared to wild-type mice exemplified by a decrease in IFN-γ production by NK cells, CD4+ and CD8+ T cells (108). Indeed, Tetherin activity might improve DCs activation and MHC-II antigen presentation in acute retroviral infection *in vivo* (109). These findings further support the idea that Tetherin not only functions as a restriction factor but also as a modulator of cellular-mediated immunity against retroviruses. Although the exact underlying molecular mechanism remains to be elucidated, the authors propose that Tetherin promotes the endocytosis and degradation of tethered viruses to induce an effective antiviral cellular immune response (109) (Figure 1). However, whether Tetherin-mediated virion endocytosis directly drives HIV-1 antigen degradation and presentation remains to be determined.

### TRIM5α

Tripartite motif (TRIM) proteins constitute a large family of E3 ligases—with approximately 100 known *TRIM* genes in humans (110)—implicated in many cellular processes including cell differentiation, apoptosis, autophagy, carcinogenesis, antiviral immunity, and innate signaling (111–114). TRIM proteins are defined by an N-terminal RBCC structure, which consists of an N-terminal RING E3 ligase domain (R), one or two B-box domains (B), and a coiled-coil domain (CC). Following the RBCC feature, the C-terminal domain(s) clusters the TRIM proteins into subgroups, with the PRYSPRY (or B30.2) domain being the most frequently found C-terminal domain among TRIM family members. Importantly, the PRYSPRY domain is responsible for the binding to the retroviral capsid and determines the spectrum of retroviral restriction (115–117).

Several TRIM family members have antiretroviral activity (114, 118), amongst which the *TRIM5* gene exhibits one of the strongest signatures of positive selection in the human genome as a marker of antagonistic virus-host coevolution and antiviral potency (1). The isoform α of TRIM5 (TRIM5α) displays the most potent antiviral activity and a large cross-species recognition pattern that underpins its importance in the control of primate lentiviral infections (2, 119). Of note, deletion or mutation of the TRIM5α RING domain impairs TRIM5α capacity to restrict viral infections (120, 121). TRIM5α is a cytosolic protein that functions in a cell-type specific manner (122–124) and inhibits retroviral replication in a species-specific fashion (115, 116, 122). For instance, TRIM5α proteins from Old World monkeys, such as rhesus macaques, restrict a broad range of retroviruses including HIV-1, HIV-2, N-tropic murine leukemia virus (N-MLV), and equine infectious anemia virus (EIAV). Nevertheless, they are ineffective against infection with certain SIV strains (SIV_mac_) (2, 125). Meanwhile, New World monkeys TRIM5α proteins do not generally suppress HIV-1. The exception is found in New World owl monkeys where the PRYSPRY domain of TRIM5α has been replaced by a cyclophilin A binding domain (TRIMCypA) that hampers HIV-1 infection very vigorously (113, 119, 126, 127). It is widely assumed that human TRIM5α (hTRIM5α) is unable to efficiently restrict HIV-1 although it strongly restricts N-MLV and EIAV (2, 125, 128, 129). However, recent studies indicate that changes in hTRIM5α expression levels and some genetic polymorphisms may influence the susceptibility to HIV-1 infection in human cells (122, 130–135), and may be associated with slow disease progression (133, 134, 136). Moreover, some primary HIV-1 clinical isolates harboring capsid mutations in response to CD8+ T cell pressure have been reported to be more sensitive to hTRIM5α inhibition compared to HIV-1 laboratory-adapted strains (137). Interestingly, it has been proposed that C-type-lectin-receptor-dependent uptake of HIV-1 might control the restriction by hTRIM5α, dictating protection or infection of human DCs subsets (122).

TRIM5α mediates its antiviral function through complementary activities, even though some of the mechanistic details remain to be elucidated. As a restriction factor, TRIM5α directly binds to the incoming retroviral capsid through its PRYSPRY domain, driving the premature uncoating of HIV-1 and impairing reverse transcription and genome translocation to the nucleus, thereby abrogating retroviral integration (2, 3, 138–140). This process requires the assembly of TRIM5α dimeric and multimeric structures with the HIV-1 p24 protein hexamers that constitute the capsid lattice (141–144). In addition, some authors suggest that the proteasomal machinery recruited via the TRIM5α E3 ubiquitin ligase activity may block retroviral replication (145–148). As Tetherin, TRIM5α may also act as a viral sensor by recognizing the HIV-1 retroviral capsid. TRIM5α binding to a susceptible retroviral capsid increases its E3 ubiquitin ligase activity, which catalyzes the synthesis of unanchored K63-linked poly-ubiquitin chains that activate the TAK1 kinase protein leading to downstream activation of AP-1- and NF-κB-dependent cascades (149–152), which may lead to the modulation of innate and adaptive immune responses. Although TRIMCypA lacks the PRYSPRY domain, it also activates the TAK1 pathway (149), suggesting that the E3 ubiquitin ligase activity (RING domain) is crucial for TRIM5α-mediated immune signaling. Indeed, several reports highlight the regulation of innate immunity by multiple TRIM proteins (153, 154).

Evolution studies of TRIM orthologs in various species revealed a remarkable paralleled co-evolution of genes encoding TRIM proteins and the development of the innate and adaptive immune systems (153, 155). This phenomenon may be indicative of a role of TRIM proteins in innate immunity and, directly or indirectly, in the establishment of the adaptive immune responses (153). Interestingly, in immune cells, mRNA expression profile studies of TRIMs led to the identification of fifteen TRIM proteins expressed at high levels in T and/or B cells (153). Similarly, the expression of a specific subgroup of *TRIM* genes was significantly upregulated in CD4+ T cells compared to macrophages and DCs (124). In a mouse model, deletion of TRIM30—the mouse homolog of hTRIM5α–enhanced the CD4+/CD8+ T cell ratio and, upon TCR activation, reduced NF-κB activation and IL-2 production in CD4+ T cells compared to wild-type mice (156). These data suggest that TRIM30 operates via the NF-kB pathway as a modulator of CD4+ T cells function. Notably, we recently showed that rhesus TRIM5α (rhTRIM5α) and TRIMCypA expression in HIV-1-infected cells leads to enhanced recognition and killing of infected cells by CD8+ T cells (157) (Figure 1). Our results strongly indicate that non-human TRIM5α variants play a role in restriction and increase CTL activation linking innate and adaptive immune responses in HIV-1 infection.

### Recently Discovered Restriction Factors

Although limited information is available, the possibility that recently discovered restriction factors may play a role in antiviral cellular immunity beyond viral restriction should not be excluded and be worth exploring.

In the case of the MARCH8 and MARCH2 (Membrane-Associated RING-CH 8 or 2 proteins) E3 ubiquitin ligases their enzymatic activity is critical for their antiviral function. These factors drive Env down-regulation from the plasma membrane and its intracellular sequestration to decrease Env incorporation into newly produced HIV-1 virions (15). In addition, MARCH2 was found to be upregulated upon HIV-1 infection in Jurkat and THP-1 cells promoting Env ubiquitination and its subsequent lysosomal degradation (16). These observations suggest a possible activity for MARCH2 in redirecting Env glycoproteins via the lysosomal route to antigen presentation pathways. Another example could be found in IFITMs (Interferon-inducible transmembrane proteins). IFITMs are small membrane-associated cellular factors that inhibit the replication of HIV-1 and other enveloped viruses at the entry step (13). IFITMs do not block the internalization of viruses but rather the virus-cell fusion process. Despite recent progress, the fate of incoming virions and the mechanistic details of IFITMs antiviral activity remain elusive. However, since IFITMs are localized in endosomes and lysosomes, it is tempting to speculate that they might be involved in targeting viruses for vesicular degradation and MHC-II loading. Indeed, the N-terminal domain of IFITM3 contains a Tyrosine Motif responsible for its endocytosis and localization in endocytic vesicles and lysosomes (158); meanwhile, the C-terminal motif of IFITM1 favors its localization in LAMP1-positive lysosomes (159). Remarkably, using the murine CMV (MCMV) model of infection, it has been shown that although IFITM3 does not restrict MCMV replication, IFITM3 deficiency leads to an impairment of cellular immunity most likely due to an unbalanced release of cytokines that drive lymphopenia including NK cell death and T cells depletion (160). Besides, in the course of influenza virus infection in mouse, IFITM3 expression in lung resident memory CD8+ T cells facilitated their survival and protection from viral infection during subsequent exposures (161). Overall, whether IFITMs also tag HIV-1 and other viruses to degradation and viral antigens production for antigen presentation and activation of T cells remains to be determined. Further insights may improve our understanding of these less characterized restriction factors, and others such us MX-2, SERINC3/5, and Schlafen 11, and their plausible function in the activation of antiviral cellular immunity.

Taken together, restriction factors may participate in shaping antiviral immunity working as a link between intrinsic cellular defenses of innate immunity and adaptive immune responses to control HIV-1 infection (Table 1 and Figure 1). Accumulating evidence suggests a connection between restriction factors and cellular degradation pathways such as the ubiquitin proteasome system, and the autophagy and endocytic routes, which may establish a framework to shape HIV-1-specific cellular immunity. In the following section, we will focus on recent studies suggesting a fine-tuned interplay between the four canonical restriction factors (A3G, SAMHD1, Tetherin, and TRIM5α) and the protein degradation pathways for the production of class I and class II viral antigens to activate HIV-1-specific T cell immunity.

**Table 1:** Restriction factors and their impact on antiviral cellular immunity.

**Restriction factor**	**Impact on antiviral cellular immunity**	**Virus**	**References**
APOBEC3G	Enhances the recognition of HIV-1 infected T cells by NK cells through upregulation of NKG2D-activating ligands	HIV-1	(46)
	Enhances the ability of HIV-1 infected T cells to activate CTL recognition	HIV-1	(50)
	Increases the production of MHC-I viral antigens in DCs favoring CTL activation		(52)
SAMHD1	Vpx-mediated SAMHD1 depletion in HIV-1 infected DCs increases viral antigen presentation leading to the activation of HIV-1-specific CTL responses	HIV-1	(80)
	Prevents virus-specific CD8+ T cell responses *in vivo*	Lentivirus	(78)
	Reduced induction of SAMHD1 in DCs from Elite Controllers induces HIV-1-specific CTL responses	HIV-1	(81)
TETHERIN	Enhances the susceptibility of HIV-infected cells to elimination by ADCC	HIV-1	(105, 106)
	Promotes NK cell, CD4+ and CD8+ T cell responses against retrovirus infection *in vivo*	Friend retrovirus	(108)
	Improves DCs activation and MHC class II antigen presentation via Tetherin-mediated virion endocytosis	Friend retrovirus	(109)
TRIM5α	rhTRIM5α and TRIMCypA improve activation of HIV-1-specific CD8+ T cell responses	HIV-1	(157)

## Restriction Factors and Protein Degradation Pathways

### The Ubiquitin-Proteasome System (UPS)

The UPS participates in the ubiquitin-dependent, and occasionally independent, degradation of cellular proteins regulating antigen processing, transcriptional modulation, signal transduction, among other cellular processes (162). Notably, in the context of HIV-1 infection, the UPS aids in the degradation and removal of viral proteins. Also, the UPS enhances the ubiquitination of cellular factors that recognize viral structures allowing the formation of high molecular complexes directed for proteasomal degradation. Besides, the UPS contributes to the regulation of signaling molecules involved in the activation of innate immunity to combat the infection (163). However, it is well-known that HIV-1, as well as many other viruses, subvert the UPS machinery to favor viral replication and escape from host immune surveillance (163–165). Indeed, HIV-1 manipulates the UPS to: (i) increase viral protein expression levels, stability, and activities by posttranslational modifications such as ubiquitination; (ii) recruit cellular E3 ligases to redirect antiviral proteins for proteasomal degradation; and (iii) to suppress the expression of host antiviral genes by controlling the activity of cellular transcription factors such as AP-1, NF-κB, and STATs, among others (68, 163, 166).

In general, in infected cells, proteins present in the cytoplasm are degraded by the UPS generating short peptides. These epitope precursors are then translocated in the endoplasmic reticulum (ER) through the action of a specific transporter associated with antigen processing (TAP) and further trimmed by ER-resident amino-peptidases to generate epitopes. These epitopes are loaded onto nascent MHC-I molecules to be displayed on the cell surface for their recognition by epitope-specific CD8+T cells. The connection between restriction factors and the UPS degradation machinery for cellular immunity is clearly exemplified by the A3G protein. A3G mediates G-to-A deamination in the HIV-1 genome promoting the integration of hypermutated provirus. Although the resultant proviruses fail to produce novel infectious particles, they can express truncated or aberrant proteins that undergo UPS-dependent degradation providing epitopes for the loading of MHC-I molecules and contributing to the activation of HIV-1-specific CTLs (50) (Figure 1). These A3G-derived HIV-1 peptides constitute a previously unrecognized source of HIV-1 antigens (50, 56). Numerous studies have characterized the involvement of the UPS in HIV-1 protein proteolysis and MHC-I-restricted antigen presentation (51), for instance by targeting to degradation HIV-1 Env protein lacking the signal sequence for translocation to the ER (167) or ubiquitin-tagged HIV-1 Nef and Gag proteins (167, 168). Remarkably, in HIV-1-infected cells, following interactions with Vif, A3G is poly-ubiquitinated and hence constitute itself a substrate for UPS-mediated degradation (38), providing as a consequence epitopes for MHC-I presentation. Indeed, A3G (and A3F)-specific CTL responses have been observed in both HIV-1-infected patients and SIV-infected rhesus macaques (169). Therefore, presentation of A3G-derived epitopes might also be a hallmark of HIV-1-infected cells targeted by the adaptive immune response.

In the case of TRIM5α, as well as other TRIM family proteins, the UPS is essential for its antiviral activity, recruiting viral components for proteasomal-mediated degradation. TRIM5α has E3 ubiquitin ligase activity and can be auto-ubiquitinated and rapidly degraded in a proteasome-dependent manner in the presence of retroviral cores (147, 170). Also, it has been shown that TRIM5α interacts with the proteasome subunit PSMC2 and in infected cells co-localizes with a complex formed by proteasome subunits and HIV-1 (146, 171, 172). Intriguingly, although TRIM5α contains a RING domain capable of ubiquitination and tagging viral proteins for proteasomal degradation, no TRIM5α-mediated ubiquitination of viral proteins has yet been detected. The functional link between restriction capacity and TRIM5α-directed proteasomal degradation was confirmed in the presence of proteasome inhibitors, which prevented the HIV-1-induced degradation of TRIM5α, blocked viral uncoating, and rescued HIV-1 reverse transcription. These observations support a model in which TRIM5α restriction of incoming retroviral capsids may depend on the formation of a TRIM5α-viral protein complex promoting TRIM5α autoubiquitination and delivery of the complex to the proteasome for degradation (147, 157, 173). Interestingly, it has been recently shown that several cellular deubiquitinating-inhibitors (DIs) enhance the poly-ubiquitination of Gag proteins and, consequently, increase Gag entry into the UPS and the MHC-I presentation pathway (174). This observation could lead to speculate that TRIM5α proteins may recruit HIV-1 components to the UPS enhancing viral antigenic peptides' availability for MHC-I presentation and activation of CD8+ T cell responses (157) (Figure 1). On the other hand, TRIM5α, TRIM21, and TRIM25 poly-ubiquitination activities have been recently shown to bridge viral degradation with the activation of innate immunity (149, 175, 176).

### The Autophagy Pathway

Macroautophagy (commonly called autophagy) is a vesicular pathway of degradation that targets components residing in the cytoplasm, membranes, or nucleus for lysosomal degradation. As such, it contributes to the turnover of cytosolic organelles, lipids, and damaged or misfolded proteins. Interestingly, autophagy also modulates innate and adaptive immune responses (177–180). Indeed, autophagy regulates inflammatory responses by targeting for instance mitochondria and inflammasomes to lysosomal degradation (181). Pioneer work also revealed the implication of autophagy in the presentation of viral antigens, in particular by MHC-II molecules to CD4+ T cells (182–184). Additionally, autophagy contributes to the MHC-I-restricted presentation of nuclear antigens (185), primarily when viruses negatively interfere with the classical pathway of MHC-I presentation (186). Effectors of autophagy also participate in the recycling and trafficking of MHC-I molecules (187, 188).

Several studies demonstrated that TRIM protein family members are involved in the modulation of autophagy, both as regulators and receptors (189). For example, TRIM23 has been attributed to modulate the activity of central components of the autophagy process (190). In the context of TRIM5α, the impossibility to rescue viral infectivity in the presence of proteasome inhibitors (138, 145, 147, 148, 170, 176) suggests that the UPS might not be the only mechanism participating in the TRIM5α-mediated viral restriction process. In macrophages, it has been proposed that TRIM5α-mediated viral restriction relies on targeting viral capsids to autophagy-mediated degradation, involving direct interactions between HIV-1 viral capsid and effectors required for the formation of the autophagy initiation complex such as ULK1 and BECLIN-1 (191–193). In Langerhans cells, TRIM5α might mediate the assembly of an autophagy-activating complex targeting HIV-1 for autophagy degradation and preventing the infection of these cells. Therefore, it is tempting to speculate that TRIM5α interactions with HIV-1 could lead to autophagy-dependent processing of viral peptides and antigen presentation to T cells (Figure 1). Indeed, Blanchet et al. have shown that upon HIV-1 infection in human monocyte-derived DCs the incoming viral particles are at least partially degraded through an autophagy-dependent pathway leading to MHC-II restricted presentation of HIV-1 epitopes to CD4+ T cells (194). In contrast, it is interesting to note that in HIV-1-infected DCs, autophagy does not contribute to the presentation of MHC-II restricted HIV-1 antigens derived from *de novo* synthesized viral proteins (195). Regardless, whether TRIM5α-mediated restriction feeds the autophagy-dependent antigen presentation pathway has not been investigated thus far. Also, note that in macrophages discordant results were reported showing no effect of the depletion of key autophagy effectors (Beclin1, ATG5, and p62) using siRNA or CRISPR-Cas9 knockout on hTRIM5α-, rhTRIM5α-, or TRIMCypA-mediated restriction (196). These discrepancies potentially rely on the complementary or redundant roles played by the UPS and the autophagy machinery in protein degradation (197, 198). Therefore, it might be worth studying side by side the contribution of proteasome- and autophagy-mediated TRIM5α retroviral restriction in various cell types.

### The Endo-Lysosomal System

The endo-lysosomal system is comprised of early and late endosomes and lysosomes allowing the trafficking of plasma membrane components and macromolecules destined to lysosomal degradation. The endo-lysosomal pathway is essential for plasma membrane protein turnover and other cellular processes such as antigen presentation or receptor-mediated cell signaling. It relies on the internalization of endocytosed proteins that are initially transported to early endosomes. Then, these proteins are either recycled back to the plasma membrane and the trans-Golgi network or sequestered into early endosomes. Once early endosomes mature into late endosomes, in a process regulated by the ubiquitin ligase RING finger protein 26 (RNF26) (199), they fuse with lysosomes for cargo protein acidification-mediated degradation. Tagging proteins with ubiquitin also allows the direct internalization of proteins toward lysosomal degradation in a process mediated by the endosomal-sorting complex required for transport (ESCRTs)-machinery (200, 201).

In the case of Tetherin, its restriction activity leads to the accumulation of HIV-1 nascent virions at the cell membrane of infected cells. Intriguingly, the fate of tethered virions is not clear thus far. While some studies suggested that retained virions may trigger cell-to-cell spread (202), others contradicted these findings (203, 204). Of note, the N-terminal cytoplasmic tail of human Tetherin not only promotes the activation of the NF-kB signaling cascade but also serves as a binding site for clathrin-mediated endocytosis and degradation of tethered virions (205). Based on this knowledge, work from Li et al. proposed a model where Tetherin-mediated endocytosis may induce specific cell-mediated immune responses against retroviral infection, although the exact mechanism is still unknown (109). Tetherin's restriction activity in retrovirally infected antigen presenting cells, such as macrophages and DCs, prevents the egress of virions from the plasma membrane. Since Tetherin's ability to enhance NK cell responses has been linked to its endocytic function, tethered virions in pDCs could be internalized in endosomes and recognized by TLR3 for the activation of NK cells (108). Moreover, the endo-lysosomal degradation of viral particles may result in increased viral peptide availability for MHC-II antigen presentation to activate CD4+T cell-mediated responses. This data would be in agreement with the mechanism observed in DCs, where DC-SIGN-mediated endocytosis of virions targets HIV-1 antigens to late endosomal/lysosomal compartments leading to MHC-II-restricted antigen presentation (206). In fact, *ex vivo* infected DCs from Tetherin wild-type mice display higher MHC-II expression and more potent stimulation of virus-specific CD4+ T cells, NK cells, and cytokine production compared to Tetherin knockout mice (207). Furthermore, Tetherin enhances CD8+ T cell responses against Friend retrovirus infection *in vivo* by antigen cross-presentation to MHC-I (108) (Figure 1).

Overall, although it remains to be firmly established, a fine-tuned interplay between restriction factors and protein degradation pathways is probably of great benefit for both the initiation of innate and adaptive immune responses, in particular to promote viral antigen presentation for the activation of virus-specific CD4+ and CD8+ T cell responses.

## Restriction Factors-based Therapeutics

The implementation of combined antiretroviral therapy (cART) has been a fundamental breakthrough in the treatment of HIV-1 infected individuals. cART maintains viral loads to undetectable levels, limits the occurrence of viral resistance, and drastically reduces the morbidity and mortality rate in HIV-1 infected individuals. However, cART does not eliminate the virus from the organism and patients need to adhere to life-long therapy, which entails drug-related side effects. In addition, treatment interruption results in a rapid viral rebound due to the presence of a latent HIV-1 reservoir (208, 209). In light of the studies reviewed here, the development of restriction factor-based therapeutics could provide alternative therapeutic avenues to control HIV-1 by synergizing intrinsic cellular sensing and activation of antiviral immunity.

### Type I Interferon Therapies

Therapeutic approaches to efficiently enhance the expression and restriction activities of A3G, SAMHD1, Tetherin, and TRIM5α have been proposed to control HIV-1 replication. Restriction factors are generally encoded by IFN-inducible genes, so the use of type I IFN (IFN-I) is being investigated to increase their expression and promote cellular protection from infection. For instance, IFN-α significantly induces A3G expression in human primary resting CD4+ T cells, macrophages, DCs, and endothelial cells in the brain restricting HIV-1 infection (210–212). Overexpression of Tetherin upon IFN-α stimulation overcomes Vpu-mediated antagonism and decreases HIV-1 virion release *in vitro* (213). Similarly, IFN-I treatment has been reported to regulate SAMHD1 function (214, 215) in a cell-type specific manner (216). The viral restriction imposed by SAMHD1 and induced by IFN signaling is exerted primarily through its activation via dephosphorylation. Thus, SAMHD1 dephosphorylation is *per se* a pharmacological target of IFN-I treatment. IFN-α also increases rhTRIM5α expression in rhesus monkey cells and hTRIM5α in human cells, enhancing their ability to restrict HIV-1 and N-MLV, respectively, (217).

A randomized controlled clinical trial evaluated the effect of IFN-α on HIV-1 viremia in early infected individuals, showing a significant decrease in HIV-1 load in IFN-α treated patients (218). However, only few studies have analyzed the effects of IFN-α treatment on the expression of restriction factors *in vivo* and the relevance of these factors to control HIV-1 infection. In rhesus macaques, intramuscular IFN-α2 administration upregulated the expression of IFN-stimulated genes, including restriction factors, and delayed systemic SIV infection. Nevertheless, prolonged IFN-α2 treatment caused IFN desensitization and decreased antiviral gene expression, enabling infection to progress thus highlighting the importance of the timing of IFN-induced innate responses (219). In ART-naïve HIV-1/HCV co-infected patients, IFN-α/ribavirin therapy suppressed HIV-1 viremia which correlated with overexpression of IFN-induced A3G, A3F, and Tetherin in patient-derived CD4+ T cells (220, 221), indicating that IFN-induced expression of restriction factors has a direct impact in inhibiting viral replication. Currently, the use of IFNα-2 is being tested in clinical trials for HIV-1 infected individuals (ID: NCT03588715; ID: NCT02227277). Besides, other IFN-α subtypes, such as IFNα-14, have shown potent antiviral efficacy by inducing the expression of some restriction factors in a humanized mice model (222). Although the stimulation of restriction factors by type I IFNs might be tempting as an experimental approach to block HIV-1, prolonged exposure to type I IFNs has been associated with persistent immune activation and disease progression (223).

### Specific Targeting by Small Molecules

Strategies to develop inhibitors or antagonists targeting HIV-1 proteins involved in counteracting restriction factors are currently being investigated with the common goal of enhancing their suppressive activity against HIV-1. For example, structural interactions between Vif-A3G and Vpu-Tetherin were used as a guide for the design of small molecules to block HIV-1 replication (224–227). Screening of compounds that inhibit Vif-A3G interaction led to the discovery of several small molecules that protected A3G from Vif-dependent degradation (228, 229) or disrupted the assembly of the Vif-ubiquitin ligase complex (230–232). A small molecule interfering with Vpu-Tetherin association that enhances Tetherin expression and potentiates its restriction capacity has also been described (225). Although a matter of debate (233), Vpu has been reported to form ion channels that might be involved in blocking Tetherin-mediated viral restriction (234, 235). Therefore, the ion channel structure formed by Vpu could be bait for the development of new antiviral agents to successfully inhibit Vpu activity increasing Tetherin's antiviral function (236).

Small molecules mimicking Vpx action and blocking SAMHD1 to induce innate immune signaling and increasing the presentation of viral antigens by DCs are also under investigation (78, 80, 237–239). Interestingly, multiple tyrosine kinase inhibitors (240) and cyclin-dependent kinase (CDK) inhibitors (241–243) impede SAMHD1 phosphorylation, promoting its antiviral activities. In particular, the CDK4/6 inhibitor Palbociclib potently restricts HIV-1 reverse transcription by maintaining SAMHD1 constantly activated (243). In addition, inhibitors of the Protein Kinase C theta have been proposed as adjuvants of antiretroviral therapy because of their effect on SAMHD1 activation and the consequent reduction of HIV-1 replication (244). Also note that topoisomerase inhibitors and chemotherapeutic drugs, albeit indirectly, trigger SAMHD1 antiviral functions by inducing DNA damage, therefore suppressing HIV-1 infection and limiting the size of the viral reservoir (245). Moreover, small compounds mimicking TRIM5α capsid interactions could be designed to block retroviral infection in the early steps of the HIV-1 replication cycle (246). In these lines, small molecules and peptides as HIV-1 capsid inhibitors are currently being explored as a new family of antiretroviral drugs (247).

A better identification and mapping of protein-protein surfaces of interactions between restriction factors and their respective antagonizing HIV-1 proteins could guide the design of small molecules for novel therapeutic interventions.

### Gene Editing: the Potential of Genetically Engineered TRIM5α Proteins

Although potential therapeutic strategies for HIV-1 infected individuals involving A3G, SAMHD1, or Tetherin have been studied (248), all of them tackle HIV-1 replication in later entry, post-reverse transcription, or post-integration events. Also, the antiviral potency of A3G and Tetherin is limited by Vif and Vpu proteins, respectively, (9, 10, 31, 37, 86, 88, 249, 250). In contrast, TRIM5α restricts replication immediately after entry and prior to integration. Of note, to date no HIV-1 accessory proteins are capable of antagonizing hTRIM5α activity (251). These unique features of TRIM5α activity make genetically engineered TRIM5α-based proteins promising candidates for the development of antiretroviral approaches and gene therapy applications compared to other restriction factors.

In recent years, several studies have supported the feasibility of exploiting TRIM5α as a target for HIV-1 gene therapy (251). For instance, a single modification of the 332 residue to proline in the PRYSPRY domain of the hTRIM5α, which increases the affinity for the HIV-1 capsid, results in a hTRIM5α variant able to strongly restrict HIV-1 infection in human cells (117, 252). Similarly, an hTRIM5α mutant generated by PCR-based random mutagenesis and functional screening also showed strong HIV-1 restriction capacity (253). Interestingly, R332G-R335G double mutations in hTRIM5α confer a degree of resistance to HIV-1 infection resembling that of rhTRIM5α. Follow-up investigations demonstrated the ability of R332G-R335G hTRIM5α mutant to potently inhibit highly diverse HIV-1 strains and clinical isolates bearing CTL escape capsid mutations in human lymphocytes (254). Although additional experiments in primary cells and including other HIV-1 variants would be required, these studies demonstrate the potential of hTRIM5α mutants as candidates for HIV-1 gene therapy. In these lines, Richardson et al. determined that hTRIM5α_R323−R332_ harboring five rhesus substitutions in the PRYSPRY domain confer protein stability and protection from HIV-1 in primary human CD4+ T cells *in vivo* (255). Chimeric proteins could also be used for instance by replacing 11 amino acids of the PRYSPRY domain of the rhesus macaque ortholog into hTRIM5α, which efficiently restricted HIV-1 infection of CCR5- and CXCR4-tropic HIV-1 clones in CD34+ cell-derived macrophages *in vitro*, and in mouse-derived thymocytes in *vivo* (256).

Previous studies have reported that the TRIMCyp fusion protein resulting from swapping the TRIM5α PRYSPRY domain by CypA retains the same function than TRIM5α, binding to the incoming retroviral capsid and impairing reverse transcription strongly restricting lentiviral infection (119, 126, 257). Neagu et al. showed that hTRIMCyp successfully inhibits CCR5- and CXCR4-tropic HIV-1 clones as well as primary isolates in CD4+ T cells and macrophages *in vitro*. Remarkably, they also found that an experimental humanized mouse model engrafted with human CD4+ T cells previously transduced with hTRIM5Cyp lentiviral vectors potently restricted HIV-1 (258). Similar to the TRIMCyp fusion construct, fusions between TRIM21 and CypA (TRIM21Cyp) elicited strong anti-HIV effects and maintained the antiviral properties of both TRIM5α and TRIM21 in human cell lines and primary human T cells (259).

One potential constrain to these therapies is the development of viral escape from TRIM5α restriction. As for cART, combining TRIM5α modified proteins with other anti-HIV strategies targeting various steps of HIV-1 replication might prevent HIV-1 evasion. For instance, Anderson et al. analyzed the efficacy of a combination anti-HIV lentiviral vector encoding a CCR5 shRNA (pre-entry), a human/rhesus macaque chimeric TRIM5α (pre-integration), and a transactivation response element (TAR) decoy (post-integration) to block productive HIV-1 infection and to inhibit the formation of novel provirus. This combination anti-HIV lentiviral vector was able to potently restrict HIV-1 infection as well as to prevent viral escape mutations (260). Later, Walker et al. evaluated the safety and efficacy of this anti-HIV lentiviral vector in CD34+ HSCs *in vivo* in a humanized murine model. Notably, they reported that mice containing transduced CD34+ HSC with the anti-HIV lentiviral vector presented selective survival advantage when challenged with both R5-tropic BaL or X4-tropic NL4-3 HIV-1 strains (261). This combination anti-HIV-1 lentiviral vector is currently in a phase I/II clinical trial (ID: NCT02797470). The exploitation of the novel CRISPR-Cas9 technology could also be a suitable tool to precisely manipulate *hTRIM5*α gene to increase the affinity of hTRIM5α for the incoming retroviral capsid and enhance antiviral potency (262, 263).

## Concluding Remarks and Future Perspectives

Cellular host restriction factors including APOBEC3G, Tetherin, SAMHD1, and TRIM5α constitute a first barrier of the intrinsic cellular response against HIV-1 and other viral infections. Recent studies highlight the role of these restriction factors as versatile actors at the interplay between innate and adaptive antiviral immunity. Restriction factors can modulate NK cells, DCs, and antiviral CD4+ and CD8+ T cell responses, although the mechanistic details are still not fully understood. The interactions between APOBEC3G, Tetherin, SAMHD1, and TRIM5α with the intracellular protein degradation pathways, including the UPS, the autophagy and endocytic pathways, may serve as a bridge to promote antiviral cellular immunity. Besides, some of the recently described restriction factors, in particular MARCH2 and IFITMs, may follow similar pathways of intracellular proteins degradation increasing the opportunities to immune regulate antiviral responses. A deeper understanding of the mechanisms underlying the immunomodulatory role of restriction factors is essential if we aim to induce early restriction and potent immune responses to control viral infections. In this way, restriction factors might offer the opportunity to design innovative therapeutic approaches to counteract retroviral replication while promoting effective antiviral cellular immunity.

## Author Contributions

All authors listed have made a substantial, direct and intellectual contribution to the work, and approved it for publication.

### Conflict of Interest Statement

The authors declare that the research was conducted in the absence of any commercial or financial relationships that could be construed as a potential conflict of interest.
